# Hypoglycemia in CDG patients due to PMM2 mutations: Follow up on hyperinsulinemic patients

**DOI:** 10.1002/jmd2.12085

**Published:** 2019-11-25

**Authors:** Hossein Moravej, Ruqaiah Altassan, Jaak Jaeken, Gregory M. Enns, Carolyn Ellaway, Shanti Balasubramaniam, Pascale De Lonlay, David Coman, Saadet Mercimek‐Andrews, Peter Witters, Eva Morava

**Affiliations:** ^1^ Neonatal Research Center Shiraz University of Medical Sciences Shiraz Iran; ^2^ Department of Pediatric Endocrinology School of Medicine, Shiraz University of Medical Sciences Shiraz Iran; ^3^ Medical Genetic Department McGill University Health Center Montreal Québec Canada; ^4^ Center for Metabolic Diseases University Hospital Gasthuisberg Leuven Belgium; ^5^ Biochemical Genetics Program Stanford University Stanford California; ^6^ Genetic Metabolic Disorders Service Sydney Children's Hospital Network Sydney New South Wales Australia; ^7^ Disciplines of Genetic Medicine & Child and Adolescent Health Sydney University Sydney New South Wales Australia; ^8^ Western Sydney Genetics Program The Children's Hospital at Westmead Sydney New South Wales Australia; ^9^ Discipline of Genetic Medicine Sydney Medical School, University of Sydney Sydney New South Wales Australia; ^10^ Reference Center for Metabolic Diseases Hospital Necker, University Paris V Paris France; ^11^ Department of Metabolic Medicine The Lady Cilento Children's Hospital Brisbane Queensland Australia; ^12^ School of Medicine University of Queensland and Griffith University Brisbane Queensland Australia; ^13^ Division of Clinical and Metabolic Genetics, Department of Pediatrics University of Toronto Toronto Ontario Canada; ^14^ Metabolic Center University Hospitals Leuven Leuven Belgium; ^15^ Department of Development and Regeneration Faculty of Medicine Leuven Belgium; ^16^ Department of Clinical Genomics Mayo Clinic Rochester Rochester Minnesota

**Keywords:** CDG, congenital disorder(s) of glycosylation, diazoxide, hyperinsulinism, hypoglycemia, phosphomannomutase 2, PMM2‐CDG

## Abstract

**Background:**

Phosphomannomutase 2 deficiency (PMM2‐CDG) is the most common congenital disorder of glycosylation (CDG). Hypoglycemia has been reported in various CDG including PMM2‐CDG. The frequency and etiology of hypoglycemia in PMM2‐CDG are not well studied.

**Methods:**

We conducted a systematic review of the literature on genetically and/or biochemically confirmed PMM2‐CDG patients who developed hypoglycemia. Prospective follow‐up information on the patients who received diazoxide therapy was collected and evaluated.

**Results:**

A total of 165 peer‐reviewed articles reporting on 933 PMM2‐CDG patients were assessed. Hypoglycemia was specifically mentioned only in 23 of these patients (2.5%). Hyperinsulinism was identified in 10 patients (43% of all hypoglycemic patients). Among these 10 patients, seven were successfully treated with diazoxide. However, most patients remained on therapy longer than a year to stay free of hypoglycemia.

**Conclusion:**

Hypoglycemia is a rarely reported finding in patients with PMM2‐CDG. Diazoxide‐responsive hyperinsulinism was found to have a good prognosis on medication in our PMM2‐CDG patients with hypoglycemia. No genotype‐phenotype correlation was observed with respect to hyperinsulinism. A prospective study should be undertaken to explore the hypothesis that hypoglycemia is underdiagnosed in PMM2‐CDG and to evaluate whether hyperinsulinism is always associated with hypoglycemia.

## 1 INTRODUCTION

Phosphomannomutase 2 deficiency (PMM2‐CDG, OMIM number: 212065) is the most common congenital disorder of glycosylation (CDG). *PMM2* mutations affect a wide range of glycosylated proteins and lipids. Most patients with PMM2‐CDG present with a multisystem disease and highly variable clinical and biochemical manifestations. Developmental disability, hypotonia, and failure to thrive are among the most frequent symptoms.^1^ Monitoring of the PMM2‐CDG patients has shown a spontaneous gradual biochemical improvement, but their clinical course has been stable during several years follow up.^2^ Since many hormone transporters and hormonal regulators are glycosylated, the endocrine system is frequently involved in PMM2‐CDG.^3^ Known glycosylated proteins include insulin‐like growth factor receptor‐binding protein 3, thyroglobulin, thyroid‐stimulating hormone, prolactin, follicle‐stimulating hormone, and luteinizing hormone, and several of these proteins affect glucose homeostasis.

In a few PMM2‐CDG patients, hypoglycemia has been reported. Although this can be severe, there is only limited data about the prevalence of hypoglycemia and its management.^4^

Upon reviewing the literature on the occurrence of hypoglycemia, and its possible causes, treatment, and outcome in PMM2‐CDG patients, we prospectively followed up on previously reported patients and their outcome.

## 2 METHODS

We performed a systematic Pubmed search using the following search terms: carbohydrate‐deficient glycoprotein syndrome OR CDG‐Ia OR congenital disorders of glycosylation type Ia OR PMM2 deficiency OR PMM2‐CDG OR phosphomannomutase2 deficiency AND hypoglycemia OR hyperinsulinism OR hyperinsulinemia.

Only genetically and/or enzymatically confirmed PMM2‐CDG patients who developed hypoglycemia and/or hyperinsulinism were included in this review.

In order to find risk factors for hypoglycemia or to figure out relations between hyperinsulinism and other manifestations of the disease, we prospectively collected data on the patients who were earlier reported with hyperinsulinism, including other possible clinical manifestations and outcome of their hyperinsulinism on diazoxide treatment. Patients were also evaluated according to the Nijmegen pediatric CDG rating scale.^5^

## 3 RESULTS

### 3.1 Literature review

Among 933 PMM2‐CDG patients, retrieved from 165 papers in Pubmed, 23 patients (2.5%) were reported to develop hypoglycemia. All of them experienced hypoglycemia in the first year of life, some already in the neonatal period.

In three patients, hypoglycemia was a leading or predominant feature.^6^, ^7^ Manifestations of hypoglycemia were the typical ones: seizures, decreased consciousness, hypotonia, and poor feeding. Their blood glucose levels were in the range of 0.5 to 2.9 mmol/L.

In 10 patients (43% of all hypoglycemic patients) hyperinsulinism was found and considered as the pathogenesis of the hypoglycemia. Their insulin levels at the time of hypoglycemia were between 8.3 and 27.7 pmol/L. Other relevant investigations, such as serum cortisol, ACTH, growth hormone, other growth factors, lactate, lipid profile, and ketone levels, were not available.

Abdominal magnetic resonance imaging (MRI) in one hyperinsulinemic patient showed a normal pancreatic anatomy.^7^ An abdominal ultrasound was performed in four patients; one normoinsulinemic patient showed a normal result and of the three hyperinsulinemic patients, two displayed a normal pancreas and one showed cystic lesions in the head of the pancreas.^6^, ^7^, ^8^

In one hyperinsulinemic patient, histology of a pancreatic biopsy showed a normal size and distribution of pancreatic islets with hypertrophic nuclei in a few beta cells.^6^

There was no significant difference between hyperinsulinemic and normoinsulinemic patients regarding the age of presentation and accompanying endocrine and other abnormalities.

Abnormal thyroid function was reported in 43% of hypoglycemic patients (n *=* 10/23). Six of them had hypothyroidism, two had decreased thyroxine levels and two had decreased thyroxine‐binding globulin levels.^6^, ^7^, ^8^, ^9^, ^10^, ^11^, ^12^

Abnormal fat pads were reported in 11 of 23 patients. Six of them were hyperinsulinemic and five were normoinsulinemic.^7^, ^8^, ^9^, ^10^, ^11^, ^13^, ^14^, ^15^

As shown in Table 1, 7 of 10 patients with hyperinsulinism were successfully treated with oral diazoxide. One patient presented with refractory hyponatremia as a side effect of diazoxide. Normal glucose level in this case was achieved after subtotal pancreatectomy.^6^ The therapeutic approach in two other hyperinsulinemic patients was not mentioned.

**Table 1:** Hyperinsulinemic phosphomannomutase 2 deficiency patients responding to diazoxide: multisystem involvement at presentation and on follow‐up

Authors	Brain		Heart		Gastrointestinal (GI) tract and liver	
	Presentation	Follow‐up	Presentation	Follow‐p	Presentation	Follow‐up
Arnoux et al^13^	Severe neurological features (stroke‐like episodes ….)	Not improved	Normal	Normal	Normal	Ascites
Coman et al^14^	Colpocephaly, prominent supratentorial and extra‐axial spaces, hypoplastic corpus callosum, cerebellar hypoplasia	Died at 3 weeks	Hypertrophic cardiomyopathy	Tamponade	GI dysmotility, food intolerance, protein losing enteropathy	Not improved
Enns et al^9^	Hypotonia	Not improved	Hypertrophic cardiomyopathy	Not improved	GER, GI dysmotility	Not improved
Shanti et al^7^ case 1	Axial hypotonia	Axial hypotonia	Cardiac tamponade	Normal	Not significant	Liver transaminitis, hypoalbuminemia
Shanti et al^7^ case 2	Normal	Normal	Normal	Normal	Normal	Normal
Shanti et al^7^ case 3	Not significant	Seizures, stroke‐ like episode	Normal	Not available	Not significant	Hypoalbuminemia, acute pancreatitis, severe GI bleeding, deranged clotting factors
Teneiji et al 12	Cerebellar hypoplasia	Not improved	Pericardial effusion	Not improved	GI dysmotility	Not improved

Abbreviation: GER, gastro‐esophagel reflux.

### 3.2 Follow‐up

In the patients successfully treated with diazoxide and remaining under regular clinical follow‐up, we collected data from their physicians on endocrine, neurological, cardiovascular, hepatic, and gastrointestinal functions and compared these data to those before the start of diazoxide treatment.

All patients who required diazoxide to control their glycemia, received the drug for more than a month. As depicted in Table 1, a wide spectrum of multisystem involvement was found. Some patients had severe progressive neurologic, cardiovascular, hepatic, or gastrointestinal involvement. Others had mild involvement of different systems. One patient had also polyhydramnios, skeletal dysplasia, nephrotic syndrome, thrombocytopenia, and ichthyosis.^14^ On the other hand, in one patient, hyperinsulinism was a predominant problem, and he did not show severe multisystem involvement at presentation neither on follow‐up.^7^

The outcome of the patients was also different. As shown in Table 2, hypoglycemia was transient in three patients and permanent in others. However, in all patients with transient hypoglycemia, the duration of treatment with diazoxide has been larger than 1 year. Four patients died in infancy^8^, ^10^, ^16^, ^17^ but six others survived with different degrees of organ involvement.

**Table 2:** Management and outcome of hyperinsulinemic phosphomannomutase 2 deficiency (PMM2‐CDG) patients

Authors	BS (mmol/l)	Insulin level (pmol/l)	Duration of hypoglycemia (Permanent/transient)	Duration of treatment	Outcome (Death/alive)	PMM2 molecular genetic testing
Arnoux et al^13^	2.7	8.3	Transient	24 months	Alive	p.24delC/p.P113L
Coman et al^14^	0.5	27.7	Permanent	Until the end of life	Died at 3.5 weeks	p.V231 M/p.D148M
Enns et al^9^	2.2	17.4	NA	NA	NA	p.L104 V/IVS1‐1
Shanti et al^7^ case 1	2.9	33	Permanent	Ongoing	Alive	p.I132T/p.F207S
Shanti et al^7^ case 2	2.6	22.6	Transient	7 years	Alive	p.F157S/p.F157S
Shanti et al^7^ case 3	2.3	23.6	NA	NA	Died at 14 years	p.R141H/p.V231 M
Teneiji et al 12	1.7	12	Transient	15 months	Alive	p.R123Q/p.G208A

Abbreviation: BS, blood sugar.

Regarding the hypoglycemia in normo‐insulinemic patients, data about etiology, therapy, and outcome were lacking.

## 4 DISCUSSION

Previous literature showed that hypoglycemia is a rare manifestation of PMM2‐CDG, and that it can be the main presentation of the disease. Hypoglycemia was transient in some patients and permanent in others requiring prolonged medical treatment. Hypoglycemia was associated with hyperinsulinism in 43% of the patients, and in the remaining patients there was no other reported etiology to explain hypoglycemia. No genotype‐phenotype correlation was observed in patients with respect to hyperinsulinism.

Although hypoglycemia is a rare presentation in PMM2‐CDG, it is frequently reported in some other CDG, including phosphomannose isomerase deficiency (MPI‐CDG) and phosphoglucomutase1 deficiency (PGM1‐CDG). Most patients with MPI‐CDG develop hypoglycemia. It is probably underdiagnosed in MPI‐CDG where, due to the often severe gastrointestinal symptoms, moderate episodes of hypoglycemia may be compensated by frequent enteral or parenteral nutrition.^18^ Hyperinsulinism is a known etiology of hypoglycemia in MPI‐CDG. Mannose treatment can improve hyperinsulinism in those patients because it bypasses the enzymatic defect and thus corrects its consequences.^19^

Most patients with PGM1‐CDG also develop hypoglycemia which seems multifactorial: hyperinsulinism, inappropriate counter‐regulatory hormonal response (low cortisol), impaired glucose release from glycogen during fasting and malnutrition.^20^ In rare cases, diazoxide therapy was required, but most of the patients responded to conservative treatment.^21^ Galactose therapy reduces the frequency of the hypoglycemic episodes in most PGM1‐CDG patients, avoiding anti‐hypoglycemic drugs (22). However, due to abnormal glycogenolysis in PGM1 deficiency, frequent feedings are needed to prevent hypoglycemia.^23^

Other CDG in which hypoglycemia has been reported are DOLK‐CDG, ALG6‐CDG, ALG3‐CDG, and ALG12‐CDG. So far, hypoglycemia seems to have been reported only in CDG‐I, not in CDG‐II. According to pathophysiology, CDG‐I there is associated with N‐glycosylation defect, but CDG‐II has a defect in O‐glycosylation.^24^, ^25^, ^26^, ^27^

The pathophysiology of hyperinsulinism in CDG has not been defined clearly. All hyperinsulinemic patients who received diazoxide in our cohort have been reported to respond well. The mechanism of function of diazoxide is not fully understood. It acts directly on the beta cells of the pancreas. It opens the K_ATP_ channel and inhibits beta cells to secrete insulin.^28^ Diazoxide responsiveness of patients with PMM2‐CDG suggests that impaired function of K_ATP_ channels could be a cause of hyperinsulinism in this disease.

K_ATP_ channels consist of sulfonylurea receptors (SUR) associated with inward rectifier Kir6.x. SUR glycosylation is very important for proper trafficking and surface expression of K_ATP_ channels.^29^ On the other hand, impaired insulin receptor function may also affect glucose homeostasis in these patients. The insulin receptor consists of two extracellular α subunits and two transmembrane β subunits. Both subunits are glycosylated: the α monomers each carry 13 N‐glycans and the β monomers four N‐glycans. The β monomers carry moreover six O‐glycans. It is not known whether the insulin receptor glycosylation is affected in CDG (30).

The role of other causes of hypoglycemia, like abnormal counter‐regulatory hormone response and malnutrition in PMM2‐CDG has not been well defined.

In addition to hyperinsulinism, hypoglycemia may be caused by other reasons.

It is not clear why hypoglycemia is less common in PMM2‐CDG than MPI‐CDG and PGM1‐CDG. The reason could be underdiagnosis, as signs and symptoms of hypoglycemia are non‐specific, and therefore, it might remain undiagnosed especially in patients with complicated neurological deficits.

As a conclusion, glycemia and insulinemia should be evaluated systematically and repeatedly in PMM2‐CDG patients to explore the hypothesis that hypoglycemia is underestimated in PMM2‐CDG, and to evaluate whether hyperinsulinemia is always associated with hypoglycemia.

It may be suggested that blood sugar be measured periodically, especially during episodes of illnesses in PMM2‐CDG patients. Patients or their parents should also be educated about symptoms of hypoglycemia.

## CONFLICT OF INTEREST

The authors declare no potential conflict of interest.

## AUTHOR CONTRIBUTIONS

H. M. conception and design, analysis and interpretation of data, drafting the article or revising it critically for important intellectual content. R. A.: analysis and interpretation of data, drafting the article or revising it critically for important intellectual content. J. J.: analysis and interpretation of data, drafting the article or revising it critically for important intellectual content. G. M. E.: analysis and interpretation of data, revising article critically for important intellectual content. C. E.: analysis and interpretation of data, revising article critically for important intellectual content. S. B.: analysis and interpretation of data, revising article critically for important intellectual content. P. De L.: analysis and interpretation of data, revising article critically for important intellectual content. D. C.: analysis and interpretation of data, revising article critically for important intellectual content. S.t M.‐A.: analysis and interpretation of data, revising article critically for important intellectual content. P. W.: analysis and interpretation of data, revising article critically for important intellectual content. E. M.: conception and design, analysis and interpretation of data, drafting the article or revising it critically for important intellectual content.

## ETHICAL APPROVAL STATEMENT

This article does not contain any studies with human or animal subjects performed by any of the authors.

## References

[1] Péanne R, de Lonlay P, Foulquier F, et al. Congenital disorders ofglycosylation (CDG): Quo vadis? Eur J Med Genet. 2018;61(11):643‐663. 10.1016/j.ejmg.2017.10.012 29079546

[2] Witters P , Honzik T , Bauchart E , et al. Long‐term follow‐up in PMM2‐CDG: are we ready to start treatment trials? Genet Med. 2019;21(5):1181‐1188. 30293989 10.1038/s41436-018-0301-4

[3] De Lonlay P , SetaN BS , et al. A broad spectrum of clinical presentations in congenital disorders of glycosylation I: a series of 26 cases. J Med Genet. 2001;38:14‐19. 11134235 10.1136/jmg.38.1.14 PMC1734729

[4] Wolthuis David FGJ , Van Asbeck EV , Kozicz T , et al. Abnormal fat distribution in PMM2‐CDG. Mol Genet Metab. 2013;110:411‐413. 24063868 10.1016/j.ymgme.2013.08.017

[5] Achouitar S , Mohamed M , Gardeitchik T , et al. Nijmegen paediatric CDG rating scale: a novel tool to assess disease progression. J Inherit Metab Dis. 2011;34(4):923‐927. 21541726 10.1007/s10545-011-9325-5 PMC3232068

[6] Böhles H , Sewell AC , Gebhardt B , Reinecke‐Lüthge A , Klöppel G , Marquardt T . Hyperinsulinaemic hypoglycaemia‐leading symptom in a patient with congenital disorder of glycosylation Ia (phosphomannomutase deficiency). J Inherit Metab Dis. 2001;24:858‐862. 11916319 10.1023/a:1013944308881

[7] Shanti B , Silink M , Bhattacharya K , et al. Congenital disorder of glycosylation TypeIa: heterogeneity in the clinical presentation from multivisceral failure to Hyperinsulinaemic hypoglycaemia as leading symptoms in three infants with phosphomannomutase deficiency. J Inherit Metab Dis. 2009;32(Suppl 1):S241‐S251. 19396570 10.1007/s10545-009-1180-2

[8] Antoun H , Villeneuve N , Gelot A , Panisset S , Adamsbaum C . Cerebellar atrophy: an important feature of carbohydrate deficient glycoprotein syndrome type 1. Pediatr Radiol. 1999;29:194‐198. 10201039 10.1007/s002470050571

[9] Enns GM , Steiner RD , Buist N , et al. Clinical and molecular features of congenital disorder of glycosylation in patients with type 1 sialotransferrin pattern and diverse ethnic origins. J Pediatr. 2002;141:695‐700. 12410200 10.1067/mpd.2002.128658

[10] Malhotra A , Pateman A , Chalmers R , et al. Prenatal cardiac ultrasound finding in congenital disorder of glycosylation type 1a. Fetal DiagnTher. 2009;25:54‐57. 10.1159/000196816 19176971

[11] Truin G , Guillard M , Lefeber DJ , et al. Pericardial and abdominal fluid accumulation in congenital disorder of glycosylation type Ia. Mol Genet and Metab. 2008;94:481‐484. 18571450 10.1016/j.ymgme.2008.05.005

[12] Teneiji AA , Bruun T , Sidky S , et al. Phenotypic and genotypic spectrum of congenital disorders of glycosylation type I and type II. Mol Genet Metab. 2017;120:235‐242. 28122681 10.1016/j.ymgme.2016.12.014

[13] Arnoux JB , Boddaert N , Valayannopoulos V , et al. Risk assessment of acute vascular events in congenital disorder of glycosylation type Ia. Mol Genet Metab. 2008;93:444‐449. 18093857 10.1016/j.ymgme.2007.11.006

[14] Coman D , Bostock D , Hunter M , et al. Primary skeletal dysplasia as a major manifesting feature in an infant with congenital disorder of glycosylation type Ia. Am J Med Genet. 2008;146A:389‐392. 18203160 10.1002/ajmg.a.32119

[15] Westphal V , Peterson S , Patterson M , et al. Functional significance of PMM2 mutations in mildly affected patients with congenital disorders of glycosylation Ia. Genet Med. 2001;3:393‐398. 11715002 10.1097/00125817-200111000-00003

[16] Rudaks LI , Andersen C , Khong TY , Kelly A , Fietz M , Barnett CP . Hypertrophic cardiomyopathy with cardiac rupture and Tamponade caused by congenital disorder of glycosylation type Ia. Pediatr Cardiol. 2012;33:827‐830. 22374380 10.1007/s00246-012-0214-y

[17] Wurm D , Loffler G , Lindinger A , et al. Congenital disorders of glycosylation typeIa as a cause of mirror syndrome. J Perinatol. 2016;27:802‐804. 10.1038/sj.jp.7211847 18034167

[18] De Lonlay P , Seta N . The clinical spectrum of phosphomannose isomerase deficiency, with an evaluation of mannose treatment for CDG‐Ib. Biochim Biophys Acta. 2009;1792:841‐843. 19101627 10.1016/j.bbadis.2008.11.012

[19] Harms HK , Zimmer KP , Kurnik K , Bertele‐Harms RM , Weidinger S , Reiter K . Oral mannose therapy persistently corrects the severe clinical symptoms and biochemical abnormalities of phosphomannose isomerase deficiency. Acta Paediatr. 2002;91:1065‐1072. 12434892 10.1080/080352502760311566

[20] Zeevaert R , Scalais E , Muino Mosquera L , et al. PGM1 deficiency diagnosed during an endocrine work‐up of low IGF‐1 mediated growth failure. Acta Clin Belg. 2016;71:435‐437. 27351072 10.1080/17843286.2016.1142043

[21] Tegtmeyer LC , Rust S , van Scherpenzeel M , et al (2014)Multiple phenotypes in phosphoglucomutase 1 deficiency. N Engl J Med; 370(6):533–42. 24499211 10.1056/NEJMoa1206605 PMC4373661

[22] Jaeken J , Péanne R . What is new in CDG? J Inherit Metab Dis. 2017;40:569‐586. 28484880 10.1007/s10545-017-0050-6

[23] Morava E , Wong S , Lefeber D . Disease severity and clinical outcome in phosphoglucomutase deficiency. J Inherit Metab Dis. 2015;38:207‐209. 25288126 10.1007/s10545-014-9769-5 PMC4344407

[24] Kranz C , Basinger AA , Gucsavas‐Calikoglu M , et al. Expanding spectrum of congenital disorder of glycosylation Ig (CDG‐Ig): sibs with a unique skeletal dysplasia, hypogammaglobulinemia, cardiomyopathy, genital malformations and early lethality. Am J Med Genet Part A. 2007;143A:1371‐1378. 17506107 10.1002/ajmg.a.31791

[25] Kranz C , Jungeblut C , Denecke J , et al. A defect in Dolichol phosphate biosynthesis causes a new inherited disorder with death in early infancy. Am J HumGenet. 2007;80:433‐440. 17273964 10.1086/512130 PMC1821118

[26] Miller BS , Hudson H , Freeze HH , et al. Pubertal development in ALG6 deficiency (congenital disorder of glycosylation type Ic). Mol Genet Metab. 2011;103:101‐103. 21334936 10.1016/j.ymgme.2011.01.016 PMC3869397

[27] Sun L , EklundEa CWK , et al. Congenital disorder of glycosylation id presenting with hyperinsulinemic hypoglycemia and islet cell hyperplasia. J Clin Endocrinol Metab. 2005;90:4371‐4375. 15840742 10.1210/jc.2005-0250

[28] Virgili N , Mancera P , Wappenhans B , et al. KATP Channel opener diazoxide prevents Neurodegeneration: a new mechanism of action via Antioxidative pathway activation. PLoS ONE. 2013;8:e75189. 24040400 10.1371/journal.pone.0075189 PMC3770693

[29] Conti LR , Radeke CM , Vandenberg CA . Membrane targeting of ATP‐sensitive potassium channel effects of glycosylation on surface expression. J BiolChem. 2002;277:25416‐25422. 10.1074/jbc.M203109200 11994306

[30] Belfiore A , Malaguarnera R , Vella V , et al. Insulin receptor isoforms in physiology and disease. An updated view. Endocr Rev. 2017;38:379‐431. 28973479 10.1210/er.2017-00073 PMC5629070

